# NLR immune receptors: structure and function in plant disease resistance

**DOI:** 10.1042/BST20221087

**Published:** 2023-08-21

**Authors:** Alexander Förderer, Jiorgos Kourelis

**Affiliations:** 1 Max-Planck-Institute of Molecular Plant Physiology, 14476 Potsdam, Germany; 2 The Sainsbury Laboratory, University of East Anglia, Norwich Research Park, NR4 7UH Norwich, U.K.

**Keywords:** NLR, phytopathology, plant biology, plant signal transduction

## Abstract

Nucleotide-binding and leucine-rich repeat receptors (NLRs) are a diverse family of intracellular immune receptors that play crucial roles in recognizing and responding to pathogen invasion in plants. This review discusses the overall model of NLR activation and provides an in-depth analysis of the different NLR domains, including N-terminal executioner domains, the nucleotide-binding oligomerization domain (NOD) module, and the leucine-rich repeat (LRR) domain. Understanding the structure-function relationship of these domains is essential for developing effective strategies to improve plant disease resistance and agricultural productivity.

## Introduction

Plants have an innate immune system that can effectively defend them against pathogens. This system depends on the activation of various immune receptors that recognize pathogen molecules, such as pathogen-secreted proteins called effectors. Effectors manipulate the host by either suppressing its immune responses or by promoting its nutrient supply to increase the pathogen's fitness. Some effectors function in the extracellular apoplastic space, while others are secreted within the host nucleocytoplasm [1].

To combat pathogens and their diverse effector repertoire, plants have evolved a large array of intracellular immune receptors, which primarily belong to the nucleotide-binding and leucine-rich repeat receptor (NLR) family [2]. Upon pathogen recognition, the activation of these NLRs is frequently linked to a localized programmed cell death, called the hypersensitive response (HR). This response is thought to help limit pathogen spread from the infection site to neighbouring cells, enabling plants to preserve overall tissue health while combating local pathogen invasions [3]. While NLR immune receptors are essential components of innate immunity across all domains of life [4–6], this gene family has significantly expanded in the green lineage, which lacks a canonical adaptive immune system. In addition, the ongoing co-evolutionary struggle between plants and their pathogens has resulted in NLRs becoming some of the most polymorphic genes within plant genomes, exhibiting significant sequence diversity and copy-number variation [7].

Canonical plant NLRs share a multidomain architecture, characterized by a central nucleotide-binding domain (NB-ARC; for nucleotide-binding adaptor shared by APAF-1, certain *R* gene products, and CED-4) involved in intramolecular regulation of the NLR, a C-terminal leucine-rich repeat (LRR) domain involved in ligand binding and intramolecular regulation, and an N-terminal domain responsible for initiating downstream signalling. The most common N-terminal domains are either Toll/Interleukin-1 Receptor (TIR) domains in TIR-NLRs or one of three sequence-unrelated but structurally and functionally conserved coiled-coil domains: the Rx-type coiled-coil (CC)-type NLRs (CC-NLRs), RESISTANCE TO POWDERY MILDEW 8 (RPW8)-type CC-NLRs (CC_R_-NLRs), and the G10-type CC-NLRs (CC_G10_-NLRs) [8].

**Figure 1.**
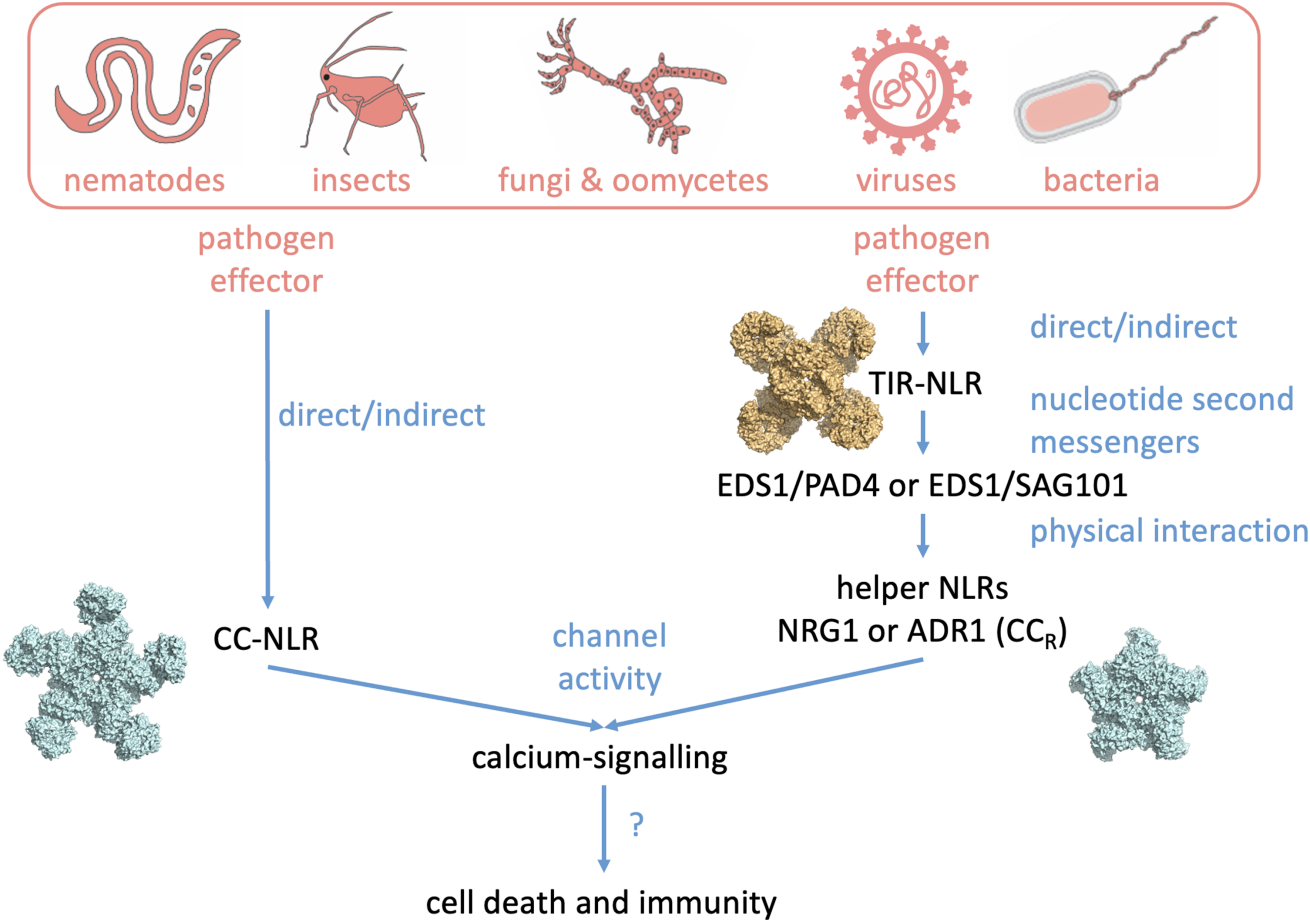
Plant NLR activation results in calcium channel activation. Model of flowering plant NLR signalling pathways. Direct or indirect effector recognition by CC-NLRs or TIR-NLRs results in resistosome formation. In the case of CC-NLRs a pentameric resistosome directly acts as a non-selective calcium-permeable cation pore at the plasma membrane. TIR-NLRs form enzymatically active tetrameric resistosome complexes which generate nucleotide second messengers which activate receptor complexes formed by the lipase-like proteins EDS1–PAD4 and EDS1–SAG101. These complexes in turn activate ADR1 and NRG1-family CC_R_-NLRs which likely form pentameric resistosome structures which also act as non-selective calcium-permeable cation pores at the plasma membrane. Physiologically, activation of these pathways often leads to a form of programmed cell death called the hypersensitive response.

Certain NLRs serve dual roles in pathogen recognition and signal transduction, whereas others have subfunctionalized into ‘sensor NLRs’ or ‘helper NLRs’, each with specialized functions; these types can operate in pairs or within more intricate networks [9]. In recent years, there has been a significant expansion in our understanding of NLR activation and the downstream signalling mechanisms involved in plant immunity (Figure 1). Upon activation, helper NLRs and NLRs with dual roles in recognition and signal transduction assemble into a higher-order ‘resistosome’ complex [10–13]. Drawing on the structures of CC-NLRs HOPZ-ACTIVATED RESISTANCE 1 (ZAR1) and Sr35, TIR-NLRs RECOGNITION OF XOPQ 1 (Roq1) and RECOGNITION OF PERONOSPORA PARASITICA 1 (RPP1), and recent advances in TIR, CC/CC_R_/CC_G10_ domain signalling, and NLR oligomerization, this review explores the key features of activated NLRs, focusing on the connection between their structure and function.

## The variable N-terminal executioner domain of plant NLRs

The N-terminal domain of flowering plant NLRs exhibits variation that reflects their phylogenetic relationships, allowing for broad classification of these proteins into four distinct subgroups: TIR-NLRs, CC-NLRs, CC_R_-NLR, and CC_G10_-NLRs [7,14]. The N-terminal domains, including TIR, CC, CC_R_, and CC_G10_, are generally considered the signalling domains that execute downstream immune responses upon NLR activation.

**Figure 2.**
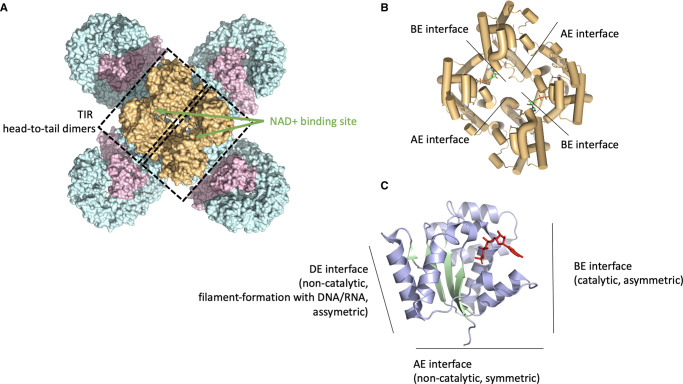
Tetrameric assembly and enzymatic activity of TIR domains upon NLR activation. (**A**) Activation of the TIR-NLR RPP1 (PDB: 7CRC) [11], results into a tetrameric assembly. Although the TIR-NLR tetramer is symmetrical, the tetrameric TIR assembly at the N-terminus of each protomer is asymmetric. TIR tetramers follow a dimer-of-dimer principle with two catalytic sites in the assembly. (**B**) Upon TIR-NLR activation the TIR domain forms a homotetrameric complex consisting of two symmetric TIR homodimers (BE interface) and asymmetric TIR homodimers (AE interface) with active centres for NAD^+^ cleavage at the asymmetric interface (RPP1; PDB: 7CRC) [11]. (**C**) In addition to the AE and BE interfaces, plant TIR domains can form filamentous assemblies using a third interface (DE), resulting 2′,3′-cAMP/cGMP synthetase activity via RNA/DNA hydrolysis.

Upon ligand recognition and NLR activation, plant TIR-NLRs can tetramerize, creating two active centres for NAD^+^ cleavage at the protomer interface formed within each asymmetric TIR homodimer (Figure 2A,B) [11,12]. The TIR domain is present across all domains of life, featuring a similar overall structure with a flavodoxin-like fold featuring central parallel β-strands surrounded by α-helices (Figure 2C) [15,16]. While some animal TIR domains function through physiological NAD^+^-depletion, plant TIR domains appear catalytically less active [16,17]. Instead, plant TIR domains function through the generation of nucleotide variants, some of which are shown to mediate downstream signalling [16–20]. These nucleotide variants generated by plant TIR domains include phosphoribosyl adenosine mono/diphosphate (pRib-AMP/ADP) and ADP-ribosylated-ADPR (di-ADPR)/ADP-ribosylated ATP (ADPr-ATP) [21]. These TIR catalyzed products bind to receptor complexes formed by the lipase-like proteins ENHANCED DISEASE SUSCEPTIBILITY 1 (EDS1) with either PHYTOALEXIN-DEFICIENT 4 (PAD4) or with SENESCENCE-ASSOCIATED GENE 101 (SAG101) [21,22]. The EDS1–PAD4 receptor complex preferentially binds pRib-AMP, leading to allosteric changes in the PAD4 EDS1–PAD4 (EP) domain and induced interaction with ACTIVATED DISEASE RESISTANCE 1 (ADR1)-type CC_R_-NLRs [22]. Meanwhile, the EDS1–SAG101 complex preferentially binds ADPr-ATP and di-ADPR promoting interaction with N REQUIREMENT GENE 1 (NRG1)-type CC_R_-NLRs through a similar allosteric mechanism [21]. The exact catalytic mechanism and the kinetics by which TIR domain activity results in the production of these final molecules and whether additional factors facilitate nucleotide conversions are currently unknown [18]. Regardless, pathogens, such as *Pseudomonas syringae*, manipulate host NAD^+^ levels to evade immune recognition. The effectors HopAM1 and HopBY achieve this by producing distinct variants of cyclic ADPR (cADPR). HopAM1 generates 3′cADPR [18], while HopBY produces the same 2′cADPR as also produced by plant TIR domains [23]. Both effectors suppress TIR-NLR mediated signalling and promote infection [23,24]. This observation suggests that the production of 2′cADPR by plant TIR domains is not sufficient on its own to induce immunity. In addition, some plant TIR domains exhibit an oligomerization mechanism distinct from resistosomes, forming filamentous assemblies with 2′,3′-cAMP/cGMP synthetase activity via RNA/DNA hydrolysis [25] (Figure 2C). Although this activity is essential for HR [25], the downstream pathway is yet to be elucidated. Finally, not all plant TIR domains signal through EDS1–PAD4 or EDS1–SAG101 modules. The ancient, conserved monophyletic group of NLRs called TIR-NB–ARC-TPRs (TNPs), found in all green plants, contain a TIR-domain phylogenetically distinct from that found in characterized TIR-NLRs [26]. TNPs likely function in an EDS1-independent manner, as they predate the origins of EDS1, PAD4, and SAG101. The function of TNPs and their TIR domains remains to be determined.

**Figure 3.**
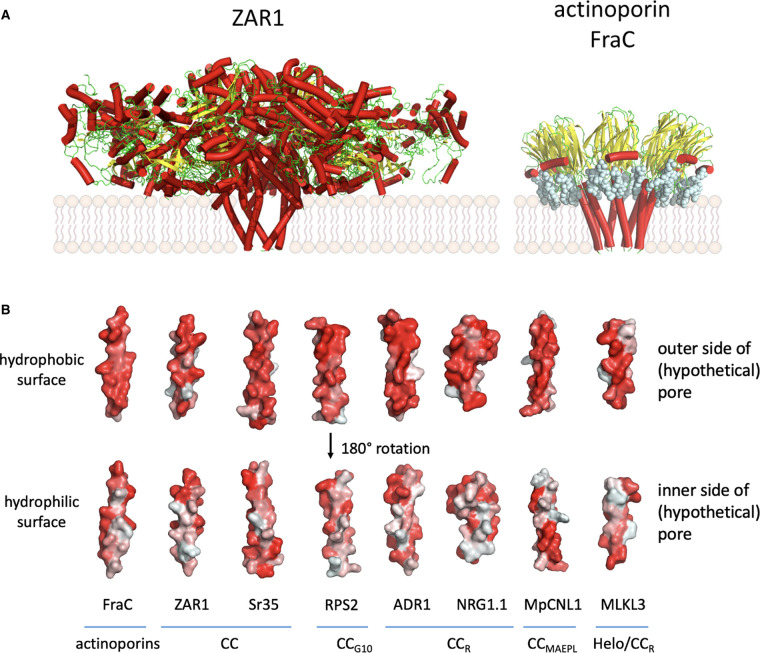
Membrane-associated functions of CC-type NLRs and their structural homologues. (**A**) Activation of CC-NLRs results in the assembly of pentameric structure, with the N-terminal CC domain forming a funnel-shaped structure with the first α-helix (α1) protruding outwards and believed to insert into the plasma membrane to create a pore (ZAR1; PDB: 6J5T) [10]. This pore is reminiscent of that formed by pore-forming toxins (PFM) of the actinoporin family (FraC; 4TSY) [28]. (**B**) The α1 of FraC and 4HB domains of CC-NLRs ZAR1 and Sr35, and the predicted α1 of CC_G10_-NLR RPS2, CC_R_-NLRs ADR1, NRG1, and the related *Marchantia polymorpha* NLR MpCNL1 [45], as well as the plant MLKL AtMLKL3 [46] have opposing hydrophobicity, with a hydrophobic exterior surface, and a hydrophilic inner surface. Surface hydrophobicity of α-helices are visualized by PyMOL using the script ‘Colour h’ ranging from grey (indicating hydrophilic) to red (indicating hydrophobic).

While referred to as a coiled-coil domain, the N-terminus of CC-, CC_R_-, and CC_G10_-NLRs can be more generally described as a four-helix bundle (4HB) domain, resembling domains found in the N-terminus of mixed lineage kinase domain-like (MLKL) proteins, Casitas B-lineage lymphoma (Cbl) proteins, heterokaryon incompatibility (HET) proteins, and the HeLo domain (for HET-s/loss-of-pathogenicity (LOP-B) protein) found in fungi [27] (Figure 3A,B) that share cytotoxic properties mediated through membrane-association activity. Despite being sequence unrelated, these 4HB domains share a similar structure and function, and are found in distinct monophyletic clades of plant NLRs.

Upon ligand recognition, CC-NLRs assemble into a pentameric structure, with the N-terminal CC domain forming a funnel-shaped structure believed to insert into the plasma membrane and create a pore. This pore is reminiscent of that formed by pore-forming toxins (PFM) of the actinoporin family (Figure 3A) [28]. Beginning with the N-terminus, the four α-helices in these domains are labelled α1, α2, α3, and α4. The α2, α3, and α4 helices make a major contribution to resistosome oligomerization through tight hydrophobic packing of CC protomers, whereas α1 does not contribute to this helical barrel arrangement and instead protrudes outward from the resistosome [10,13,29,30]. Reminiscent of the membrane insertion of actinoporin toxins, the α1 helix of CC-NLRs and other 4HB domains is thought to insert into the plasma membrane due to their opposing hydrophobicity, with a hydrophilic inner side and a hydrophobic outer side in the resistosome funnel (Figure 3B).

Both the ZAR1 resistosome and the Sr35 resistosome function as non-selective calcium (Ca^2+^) permeable cation channels [13,31]. The α1 helix of ZAR1 overlaps with a functionally conserved MADA-motif, found in ∼20% of flowering plant NLRs [32]. Specific mutations in hydrophobic residues of the ZAR1, Sr35, and NLR-REQUIRED FOR CELL DEATH (NRC)-helper NLRs MADA-motif can prevent downstream immune responses without affecting resistosome formation [13,33–35]. Moreover, negatively charged residues in the α1 helix may be crucial for the Ca^2+^ channel activity of certain, but not all, CC-NLRs [13,31,32]. While there is growing evidence for the roles of specific amino acids in channel function, a fully predictive understanding of each individual amino acid's contribution remains to be established, and structural information of a CC-NLR resistosome in a membrane context is currently lacking.

The third α-helix (α3) of CC-NLRs contains a conserved and acidic EDVID motif, named after the most prominent amino acid residues found at each position in the motif, and which interacts with basic arginine residues in the LRR to stabilize both the active and inactive form of CC-NLRs via several salt bridges [13]. CC_R_-NLRs also oligomerize upon activation, with the CC_R_ domain acting as a Ca^2+^ channel [36,37]. Similar to some CC-NLRs, negatively charged residues in the α1 helix of ADR1- and NRG1-family CC_R_-NLRs are required for Ca^2+^ channel function and cell death induction, while NRG1-family CC_R_-NLRs additionally have an unstructured extension in front of the α1 helix which is necessary for immune signalling but not for plasma-membrane association or NLR oligomerization [36,38].

The second (α2) and fourth α-helix (α4) of both CC- and CC_R_-NLRs are important for immune signalling, proper localization, and specific phospholipid binding likely determined through their surface exposure in the inactive conformation of the NLR [39]. In the active resistosome form of CC-NLRs, however, the α2 to α4 helices form a helical barrel buried at the core of the structure. Indeed, the NRC4 helper NLR localizes to a specific pathogen-induced plasma membrane structure called the extrahaustorial membrane, and this localization depends on the NRC4 CC domain [40]. However, constitutively active NRC4 does not exhibit this localization, perhaps because these lipid-binding residues are buried in the active state [40]. Similarly, emphasizing the shared features between these 4HB domains, the MLKL domain can also bind phospholipids; however, the significance of this phospholipid-binding in relation to its immune function remains unclear [41]. Structural modelling reveals that the CC_G10_ domain shares a similar four-helix bundle with CC/CC_R_-NLRs. Some CC_G10_ domains possess an N-terminal extension, which can be modified by the covalent attachment of fatty acids and which is required for localization to the plasma membrane and immune signalling [42]. As is the case for CC-NLRs, the predicted α1 helix of CC_G10_-NLRs is essential for cell death-inducing activity, and this cell death-inducing activity can be blocked by Ca^2+^-channel inhibitors, suggesting CC_G10_-NLRs like CC/CC_R_-NLRs also act as calcium-permeable channels [14,43].

Finally, non-flowering plant NLRs exhibit a greater diversity of N-terminal domains, including protein kinase domains and α/β hydrolase domains [44,45]. Whether these domains are involved in signal transduction and how oligomerization regulates this function remains to be demonstrated.

## Switching states: the NB-ARC domain's role in NLR activation and oligomerization

**Figure 4.**
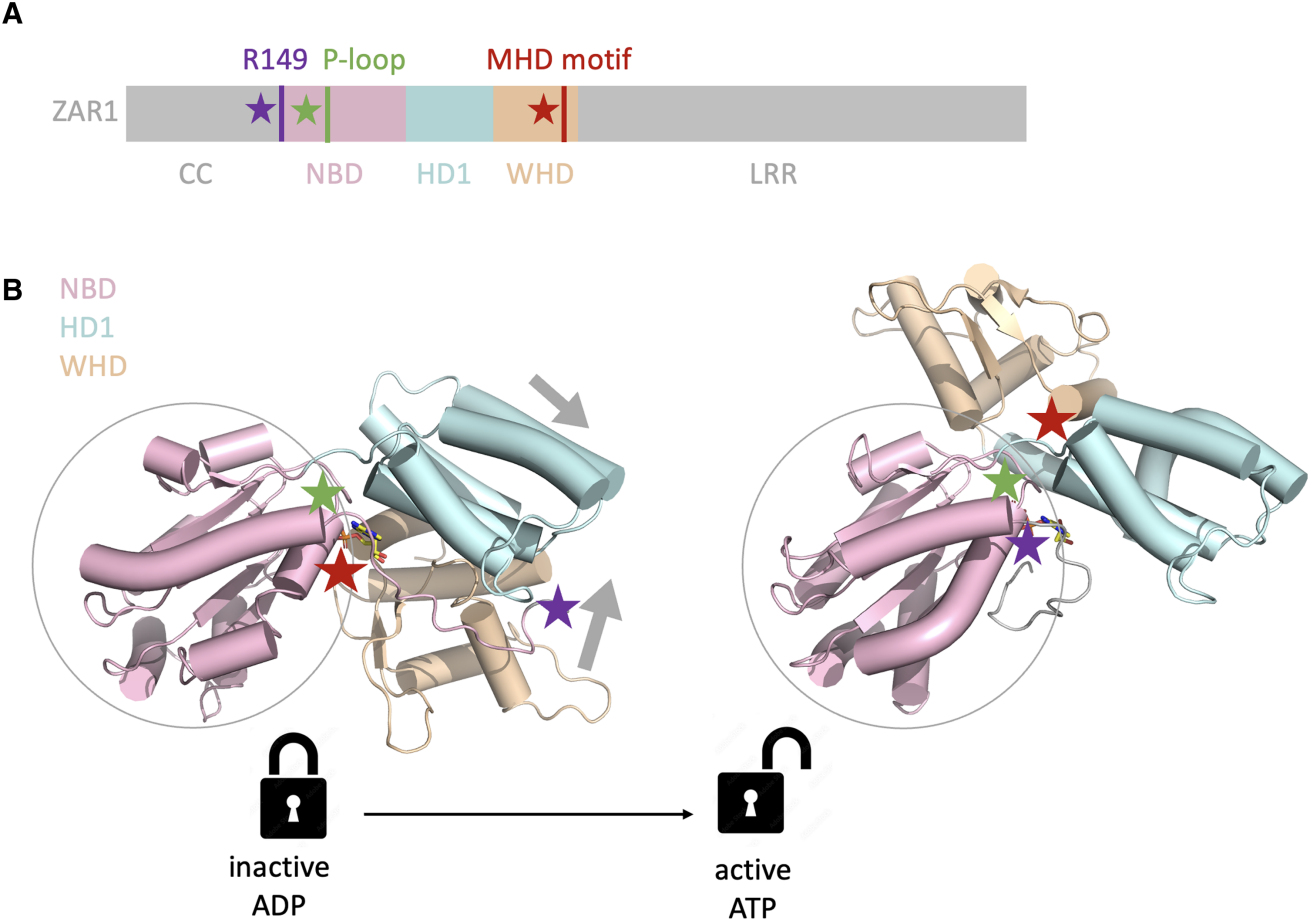
NOD module structural rearrangement during activation of ZAR1. (**A**) Relative position of important structural features (motifs, residues) and domains along the protein primary sequence of ZAR1. The NB-ARC nucleotide-binding and oligomerization domain (NOD) module is comprised of a nucleotide-binding domain (NBD; pink), helical domain 1 (HD1; cyan), and winged helix domain (WHD; orange). (**B**) The NOD module displays a large structural rearrangement between the inactive (left) and active state (right) as indicated by the arrows relative to the NBD. This rearrangement is accompanied by an exchange of ADP (yellow) for ATP (yellow) in the NBD nucleotide pocket which is partially formed by the P-loop (green star) and an interaction of ATP with ZAR1^R149^ (purple star). Concurrently, the conserved MHD motif (red star) is displaced.

The NB-ARC domain is a highly conserved feature in plant NLRs, providing a basis for their classification and function [8]. The NB-ARC domain represents a subclade of the signal transduction ATPases with numerous domains (STAND) proteins [47]. More broadly, NLRs can be defined as proteins containing a STAND family domain, referred to as a nucleotide-binding and oligomerization domain (NOD) module in this context, along with ligand-binding and executioner domains. These proteins oligomerize upon ligand recognition and activation to trigger downstream signalling through their executioner domains. Structural studies reveal that the NOD module, comprising a nucleotide-binding domain (NBD), helical domain 1 (HD1), and winged helix domain (WHD), is highly conserved across plant, animal, and bacterial NLRs [48] (Figure 4A). Although the NB-ARC domain consists of NBD, HD1, and WHD subunits, other NOD modules within the STAND family can also include an additional helical domain 2 (HD2) following these three domains [48]. These modules display similar state transitions from their inactive to their active states, indicating the conserved nature of NLR activation mechanisms across different organisms regardless of the exact identity of the NOD module, or the ligand-binding and executioner domains.

In inactive NLRs, inhibitory contacts maintain the NBD, HD1, and WHD in a closed state [29]. Activation involves conformational changes in the NBD-HD1 module relative to the C-terminal WHD (NBD hinge), as revealed by the structures of ZAR1, Sr35, RPP1, and Roq1 (Figure 4A,B) [10–13,29,30]. The activated NLR's WHD rotates away from the nucleotide-binding site, displacing the conserved MHD motif within the WHD and ZAR1^R149^ within the NBD thereby exposing the oligomerization interface. This configuration enables the NBD-HD1 surface of a protomer to interact with the NBD-WHD surface of its neighbour. The major interactions involve an HD1-WHD interface and an NBD-NBD interface. ATP/dATP, which is nested in the P-loop and a pocket in the NBD, stabilizes the active conformation of ZAR1 through direct interactions of the gamma phosphate group of ATP with ZAR1^R149^ [10]. Changes in relative position and contributions to stabilization of the active versus inactive states, as seen in ZAR1^R149^ and the MHD motif for instance, have facilitated mutation-based inactivation and autoactivation of NLRs, respectively (Figure 4A,B). Although the ADP-ATP switch in ZAR1's NB-ARC domain is necessary for oligomerization into a pentameric resistosome structure, RPP1 forms an ADP-bound resistosome, potentially explained by a charge change in the conserved ‘TT/SR’ motif found in the HD1 [11]. Other interactions might compensate for the loss of ATP-mediated stabilization of the RPP1 resistosome, such as TIR tetramerization [11]. The linker region between the N-terminal and NBD domains also plays a role in resistosome oligomerization, and the linker length and flexibility may accommodate and reinforce the oligomeric state of the activated resistosome [12].

Upon activation, CC_R_-NLRs also oligomerize, but their exact structure and the binding interface with EDS1–PAD4 or EDS1–SAG101 remains undescribed [36,37,39]. EDS1 is not necessary for cell-death induction by autoactive mutants of CC_R_-NLRs [36], suggesting that activated CC_R_-NLRs may not need to remain bound to EDS1–PAD4 or EDS1–SAG101. Similarly, while the exact structure of activated CC_G10_-NLRs is unknown, it is likely they form resistosome complexes, and the CC_G10_-domain has the potential to oligomerize on its own [43].

Less is known structurally about the activation of paired and networked plant NLRs. The rice Pik and Pia CC-NLR pairs, as well as the Arabidopsis RESISTANT TO RALSTONIA SOLANACEARUM 1 (RRS1)–RESISTANT TO P. SYRINGAE 4 (RPS4) TIR-NLR pairs, interact prior to activation [49–51]. However, it remains to be determined whether these pairs form resistosomes, remain bound in the activated resistosome state, and how activation of the sensor NLR leads to helper NLR-dependent immune responses. In mammalian systems, the activation of paired NLRs such as NLR family apoptosis inhibitory proteins (NAIPs) and NLR family CARD-containing protein 4 (NLRC4) is better understood. Prior to activation, NAIPs are in an open conformation and interact with inactive NLRC4. Ligand binding by NAIPs triggers a 20° rotation in its WHD, leading to a steric clash with NLRC4's NBD. This ‘trans’ clash results in the activation of NLRC4 and the formation of a disc-like inflammasome [52]. It remains to be determined whether Pik, Pia, and RRS1/RPS4 pairs form heterooligomers upon activation, with both sensor and helper NLRs as part of the active resistosome. In the NRC network, composed of multiple sensor and helper NLRs, sensor NLR-dependent activation of NRC helpers results in the oligomerization of these helper NLRs. Unlike the NAIP/NLRC4 example, sensor NLRs neither form part of the activated oligomeric complex nor seem to oligomerize [34,35]. Whether NRC helper activation involves the displacement of negative regulators, such as found for the activation of the nematode *Caenorhabditis elegans* CED4 NLR [53], transient interactions with sensor NLRs, or another intermediate signal, remains to be determined.

## Decision-making: the C-terminal ligand-binding domains and other ligand-binding domains

The mechanism of pathogen detection by NLRs can be either direct or indirect. In direct recognition, NLR proteins recognize effectors through direct physical interaction without the need for additional host proteins. Indirect detection follows the guard and decoy models, where NLRs monitor the status of a host component, known as a guardee or decoy [54].

Most plant NLRs possess an LRR domain that has a distinct horseshoe shape, which differs from the elongated and sometimes twisted shape of LRR domains found in many plant cell surface receptors. This distinct architectural difference could be the result of evolutionary constraints arising from the principle of ‘form follows function’, and these constraints could be a direct consequence of the NLR activation mechanism. Both direct and indirect ligand recognition by plant NLRs appear to employ an allosteric mechanism involving a ‘steric clash’ with the NBD [13]. In this steric clash model, ligand-binding at the ascending lateral side of the LRR domain allows for NBD dislodgment, resulting in conformational changes that are stabilized by the exchange of ADP for ATP, ultimately leading to resistosome assembly. Comparisons of plant resistosome structures suggest that this mechanism is conserved. In Sr35, Roq1, and RPP1, direct effector binding displaces the NBD, while in ZAR1, NBD displacement occurs upon indirect recognition of pathogen effectors that promote interactions with receptor-like cytoplasmic kinases [10–13,29]. A similar steric clash activation mechanism which results in activation of N-terminal executioner domains is also conserved in mammalian NLRs [52], and may therefore represent a more general activation strategy across the STAND protein family. Structure-based mutagenesis and evolution-guided approaches enable the exchange of ligand recognition between related NLRs [13,55]. In principle, novel recognition specificities should be enabled by engineering the C-terminal ascending site of the LRR to result in a steric clash with the NBD upon ligand-binding. For example, identifying allelic variation in the C-terminal ascending site of Sr50 and Sr33 allows for switching recognition specificity of these receptors [55]. Combining the knowledge from effector-receptor interfaces with natural and artificial allelic variation at these sites could allow for the generation of LRR libraries as a resource for rapid resistance engineering.

The RPP1 and Roq1 structures revealed a previously unobserved domain directly following the LRR domain termed the C-terminal jelly roll/Ig-like domain (C-JID) [11,12]. The C-JID is only found in association with TIR-NLRs and is directly involved in ligand-binding and may also enhance ligand-binding to the LRR. In some paired TIR-NLRs, the C-JID might regulate intramolecular interactions [56].

Finally, plant NLRs can also possess other ligand-binding domains. For instance, the N-terminal late-blight resistance protein R1 domain (also known as the Solanaceae domain) occurs exclusively with the NB-ARC domain and might be involved in ligand-binding [57,58]. Across the kingdoms of life, NLRs have converged on different superstructure-forming repeat domains involved in ligand recognition, serving the same function as the canonical LRR domain typically found in plant NLRs. Examples of these alternative domains include WD40 repeats, such as found in the animal apoptosome-forming NLRs [59,60], and tetratricopeptide repeats (TPRs), which can be found in bacterial anti-phage Avs4 complexes [6]. Non-flowering plant NLRs display a broader diversity of potential ligand-binding domains, including ankyrin (ANK) repeats, tetratricopeptide repeats (TPRs), and armadillo (ARM) repeats, some of which are infrequently also found in flowering plant NLRs [26,44,61]. Additionally, some sensor NLRs called NLR-IDs contain noncanonical ‘integrated domains’ that can function as baits for pathogen detection [62,63]. These domains appear to have evolved through effector target domain fusion into an NLR. The sequence diversity of integrated domains in NLR-IDs is vast and is found across the diversity of plant NLRs, indicating recurrent domain acquisitions throughout plant NLR evolution [63,64]. Mutating or swapping these integrated domains can be sufficient to alter NLR specificity [65–70].

## Conclusion

In summary, the N-terminal executioner domains, the NOD module responsible for the switch between inactive and active states, and the ligand-binding domain that facilitates NLR activation all contribute to the dynamic and versatile nature of these immune receptors. A deeper understanding of these molecular mechanisms and how they evolved will pave the way for the development of novel strategies to enhance plant resistance against pathogens and improve agricultural productivity.

## Perspectives

- Understanding the structure-function relationship of plant NLRs is essential for devising efficient strategies to enhance plant disease resistance and boost agricultural productivity.
- Current understanding indicates that ligand-binding by the C-terminal LRR domain of plant NLRs induces structural rearrangements in the nucleotide-binding oligomerization domain (NOD) module, which results in resistosome formation and activation of the N-terminal executioner domains. CC-NLRs create non-selective calcium-permeable pores at the plasma membrane, while TIR-NLRs form enzymatically active resistosome complexes that indirectly activate CC_R_-NLRs to create similar pores at the plasma membrane.
- Future directions in the field will likely focus on increasing our ability to predict the specific contributions of individual amino acids to NLR function, obtaining structural information on resistosome complexes within a membrane context, and investigating the activation mechanisms of paired and networked NLRs.

## Open Access

Open access for this article was enabled by the participation of University of East Anglia in an all-inclusive *Read & Publish* agreement with Portland Press and the Biochemical Society under a transformative agreement with JISC.

## Abbreviations

ADP: adenosine diphosphate
ADR1: activated disease resistance 1
AMP: adenosine mono phosphate
EDS1: enhanced disease susceptibility 1
HD1: helical domain 1
HET: heterokaryon incompatibility
HR: hypersensitive response
LRR: leucine-rich repeat
MLKL: mixed lineage kinase domain-like
NAIPs: NLR family apoptosis inhibitory proteins
NBD: nucleotide-binding domain
NLR: nucleotide-binding and leucine-rich repeat receptor
NLRC4: NLR family CARD-containing protein 4
NOD: nucleotide-binding oligomerization domain
NRC: NLR-required for cell death
PAD4: phytoalexin-deficient 4
PFM: pore-forming toxins
RPP1: recognition of peronospora parasitica 1
RPS4: resistant to P. syringae 4
RRS1: resistant to ralstonia solanacearum 1
SAG101: senescence-associated gene 101
TIR: Toll/interleukin-1 receptor
TPRs: tetratricopeptide repeats
WHD: winged helix domain
